# Targeted Support for Nursing, Midwifery and Allied Health Professional Research Funding Within an Acute UK NHS Trust: A Brief Report

**DOI:** 10.1002/nop2.70982

**Published:** 2026-11-01

**Authors:** J. C. Menzies, H. Pluess‐Hall, C. Meek, V. Sriram, E. Swingwood

**Affiliations:** ^1^ Faculty of Nursing Education and Research Bristol Royal Hospital for Children, Bristol NHS Foundation Trust Bristol UK; ^2^ Imperial College London London UK; ^3^ Centre of Health and Clinical Research University of the West of England Bristol UK; ^4^ University of Bath Bath UK; ^5^ Adult Therapy Services Bristol NHS Foundation Trust Bristol UK; ^6^ Bristol NHS Foundation Trust Bristol UK

**Keywords:** allied health professions, capability building, capacity building, funding, midwifery, nursing, quality improvement, research, support

## 1 Introduction

### 1.1 National and Regional Strategies

Research active organisations consistently demonstrate better patient outcomes (Ozdemir et al. 2015), enhanced patient experience (Salge and Vera 2009) and improved staff satisfaction and retention (Harding et al. 2017). Recent national strategies for Nursing, Midwifery and Allied Health Professionals (NMAHPs) send a strong message that research must be embedded in everyday practice and professional decision‐making for public benefit (NHSE 2021, 2023; HEE 2022; RCN 2025). Over the past 4 years, significant investment has supported the operationalisation of these ambitions, including research delivery training for undergraduate students (Henshall et al. 2024), NIHR undergraduate internships (NIHR 2024a), toolkits for leaders to build research‐engaged workforces (NHSE 2025a) and the Multi‐Professional Practice‐based Research Capabilities Framework (NHSE 2024), which outlines research development expectations across all career stages.

In 2023, our organisation‐ University Hospital Bristol and Weston NHS Foundation Trust (UHBW) launched an NMAHP‐specific strategy aligned with the NHS Trust Research Strategy, positioning *excellence in research and innovation* as a core priority. Key goals included establishing visible clinical academic career pathways and empowering staff to participate in and lead research. With clear expectations set nationally, regionally and organisationally, this was an important moment to invest in strengthening NMAHP research engagement.

### 1.2 Research Capacity and Capability Building

Although the value of clinical academic roles is well recognised, the workforce has continued to decline and was described as in “crisis” in 2024 (Wise 2024). In response, the Office for the Strategic Coordination of Health Research (OSCHR) convened two Task and Finish groups to examine barriers and opportunities for clinical academic careers (United Kingdom Research and Innovation (UKRI) 2025a, 2025b). Frequently cited barriers include lack of time, training, and support (Dickinson et al. 2017; Avery et al. 2022; Carrick‐Sen et al. 2019; Richardson et al. 2019; Oulton et al. 2022). NIHR fellowships and awards aim to address these issues by providing protected time, structured training, and supervision (NIHR 2024b). However, NMAHPs remain underrepresented and experience lower success rates (Mulvey et al. 2022).

### 1.3 Organisational Self‐Assessment

In 2023, following publication of a Trust Research Strategy for NMAHPs, an internal review examined applications for early‐career research opportunities and found that none had been submitted. A small working group was therefore established to support delivery of the NMAHP research strategy, with a specific remit to build research capacity and capability. A key priority was developing future research leaders by increasing applications to early‐career research fellowships and awards. This paper outlines work undertaken to address this.

## 2 Method

### 2.1 Context

This work was undertaken within a large NHS organisation in England employing approximately 13,700 staff, including 8867 NMAHPs. The organisation serves a population of more than 500,000 people across 10 hospital sites.

### 2.2 Problem‐Statement

Low levels of awareness and submission to early career research funding schemes from NMAHPs.

The aim of this initiative was to increase NMAHP's awareness and understanding of regional early‐career research funding schemes and to increase applications within 18 months.

Project objectives were:

1. To quantify the number of people seeking information
2. To describe the type and amount of support provided
3. To quantify the number of developed and submitted applications, alongside application outcomes with commentary on and associated income generation.
4. To gain staff feedback on the support offered

### 2.3 Design

A Plan–Do–Study–Act (PDSA) approach was adopted to guide the improvement process. PDSA is a recognised quality improvement methodology that supports the iterative testing of change through repeated cycles of planning, implementation, evaluation and refinement, enabling continuous learning and adaptation throughout the improvement process (NHSE 2015). This approach was considered particularly appropriate for the present project because implementation involved a diverse, multiprofessional workforce with differing roles, responsibilities and clinical workflows. Consequently, a standardised, “one‐size‐fits‐all” implementation strategy was unlikely to be effective across all professional groups. In this work we describe one full PDSA cycle.

### 2.4 Plan

The project team comprised two post‐doctoral clinical academics (a nurse and a physiotherapist), supported by the Director of Allied Health Professionals (an occupational therapist) and a nurse participating in the NIHR Senior Research Leader (SRL) programme (NIHR 2024c). All four team members were healthcare professionals with responsibility for building organisational research capacity and capability and involved with working on initiatives aligned to the Trust NMAHP research strategy. In addition, the team drew on results from a local Trust survey which explored barriers to research engagement using a modified version of a validated tool (the Research Capacity and Culture Tool) (Holden et al. 2012). The survey (unpublished) identified the biggest barriers were lack of protected time, lack of knowledge and skills, and uncertainty about where to access support/guidance. Building on this, the team identified three primary drivers: (1) lack of staff awareness of funding schemes, (2) lack of staff understanding of the schemes and (3) absence of application submissions and/or success (Figure 1). In line with Quality Improvement guidance (Institute for Healthcare Improvement 2017), secondary drivers were then outlined which were specific, actionable factors that contribute to each primary driver and associated change ideas were developed as interventions or actions to address the secondary drivers.

**FIGURE 1:**
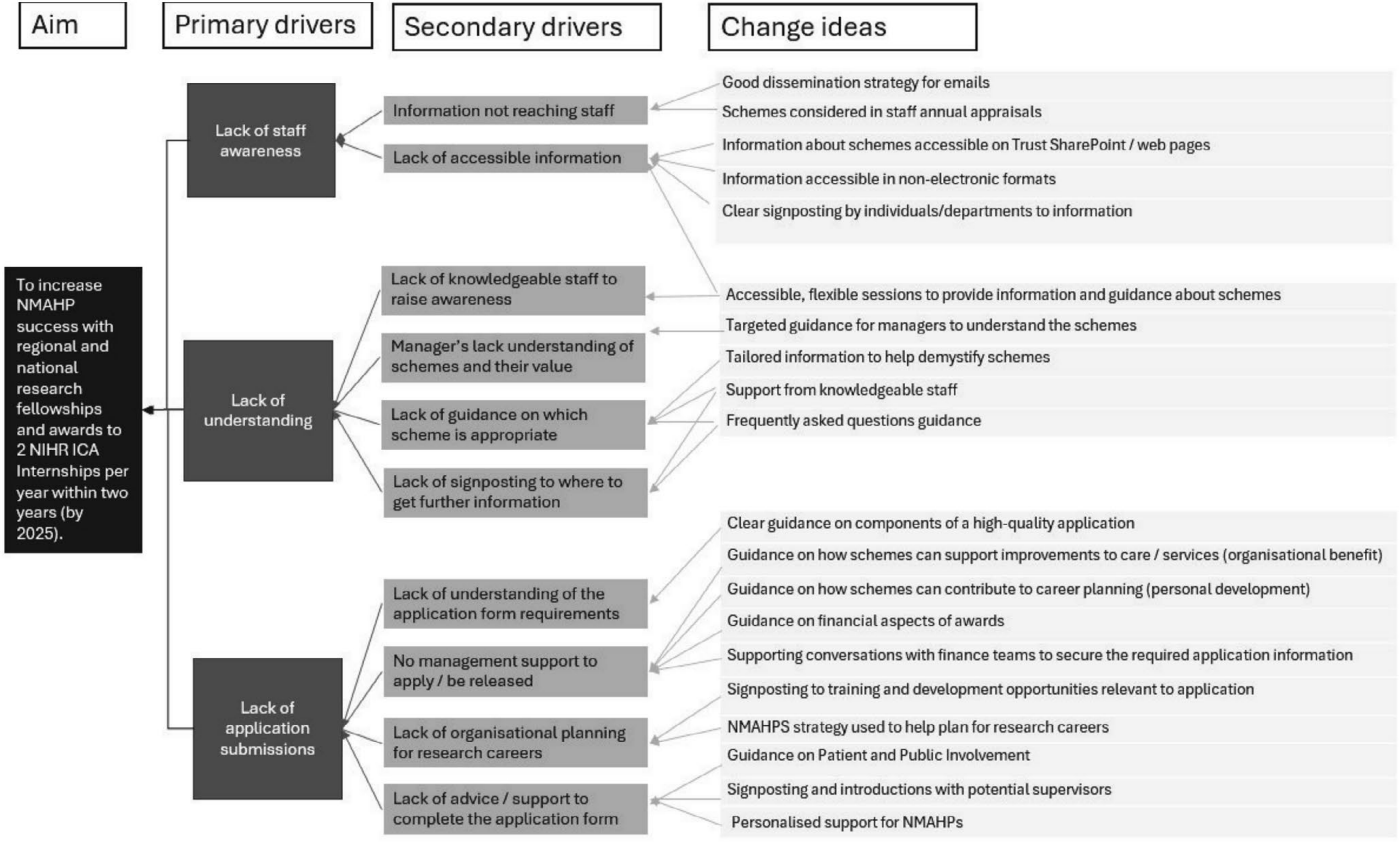
Driver diagram outlining the aims, primary and secondary drivers for the QI project to increase NMAHP research engagement. Abbreviations: NIHR ICA, National Institute of Health Research Integrated Clinical Academic (research funding stream); NMAHP, nurse, midwifery and allied health professionals.

### 2.5 ‘Do’

The intervention involved team members delivering a series of planned support sessions aligned with the launch of regional early researcher schemes. Each session, delivered either online or face to face, was hosted by at least one team member and provided the opportunity for colleagues interested in the schemes and managers to discuss the schemes' purpose and eligibility criteria, potential ideas and methods, funding requirements and applications. Promotional materials with session dates and times were circulated via Trust email lists, internal communications and the Trust Research SharePoint site.

The targeted schemes were:

1. *Personal research training award* at a local Higher Education Institution (HEI) (University of the West of England (UWE)), consisting of either a funded research methods module or a dissertation (20 Master's‐level credits). Applications required a short form and supporting CV. Each module cost £865. The scheme ran twice yearly, in May and November.
2. *NIHR Integrated Clinical Academic (ICA) Internship*, delivered regionally through a local HEI (UWE). Also offered biannually (May and November), this award provided up to £12,500 to cover training, supervision, patient and public involvement and engagement (PPIE) and research activity.
3. *Biomedical Research Centre (BRC) Fellowship*, launched in 2024 through a separate HEI (University of Bristol). Offered annually, it provided funding for supervision, training, PPIE and research activity for 1 year, with no fixed funding limit

### 2.6 Study

From the outset the team collated information about staff contact/support in an Excel spreadsheet stored on a secure shared drive. Data captured included:

1. *Number of NMAHPs seeking information (objective 1)*: Profession and frequency of contact were recorded for all clinicians engaging with support
2. *Type and amount of support provided (objective 2)*: This included method of contact, topics discussed and time spent by the team. After the first round of support sessions (May 2023), the team retrospectively reviewed records, coding independently and then together to ensure reliability. From this, we developed a coding template with seven core topics which was used prospectively from October 2023 onwards (Data S1).
3. *Submitted awards and outcomes (objective 3)*: Individuals updated the team on application submissions, interview offers (ICA Internship May and October 2023, May 2024) and submission outcomes. The team met monthly to review engagement, track support provision and ensure documentation was complete.
4. *Staff feedback (objective 4)*: Following support sessions for two funding schemes (May and October 2023), the team sought feedback from participants. As no validated tool existed, a survey was developed in Microsoft Forms using best practice guidance (Latour and Tume 2021). Survey questions were informed by previously reported barriers to NMAHP research engagement, including lack of funding (Trusson et al. 2019; Avery et al. 2022), limited support for developing research questions (Olive et al. 2021), insufficient mentorship or supervision (Trusson et al. 2019; Olive et al. 2021; Avery et al. 2022) and lack of managerial support (Richardson et al. 2019; Carrick‐Sen et al. 2019). The survey aimed to identify whether these barriers were experienced locally and if targeted support helped address them.

Participants were asked to indicate prior awareness of the schemes, the types of support accessed, outcomes of seeking advice and reasons for not submitting applications (where relevant). Likert scales measured satisfaction with support, the perceived importance of dedicated help for developing research ideas and whether further investment in such support was required. All questions were mandatory to prevent missing data (Table S2).

The evaluation survey was piloted with four research staff who had not accessed support to test clarity and functionality; minor amendments were made to introductory text to emphasise inclusivity and data anonymity. The survey was then distributed to all NMAHPs who had contacted the team, a week after submission deadlines but before outcomes were known to avoid bias. Weekly reminders were sent for 2 weeks to maximise response rates.

### 2.7 Analysis

All quantitative data were analysed descriptively. One optional free‐text question was included, and responses were analysed using content analysis (Bengtsson 2016). Illustrative quotes were identified to help provide examples.

### 2.8 Ethical Considerations

Using the NHS Health Research Authority (HRA) decision tool (NHS HRA 2023) this project was not deemed ‘research’. The project was classified as Quality Improvement (QI) and registered on the Trust QI database in line with local governance procedures. All survey responses were anonymised to protect participant confidentiality.

### 2.9 Act

Following analysis, the team completed the PDSA cycle, reviewing data related to the four objectives to understand how this support could be taken forward.

## 3 Results

We provided a mean of seven support sessions per funding call for the three schemes May 2023–May 2024. A total of 26 sessions were offered for the November 2024 funding call, because the scheme changed (with an expanded remit beyond purely clinical academic/practitioner development) and launched with a later deadline of mid‐December. In addition, the BRC scheme also launched, so the core team felt more sessions were required (Figure 2).

**FIGURE 2:**
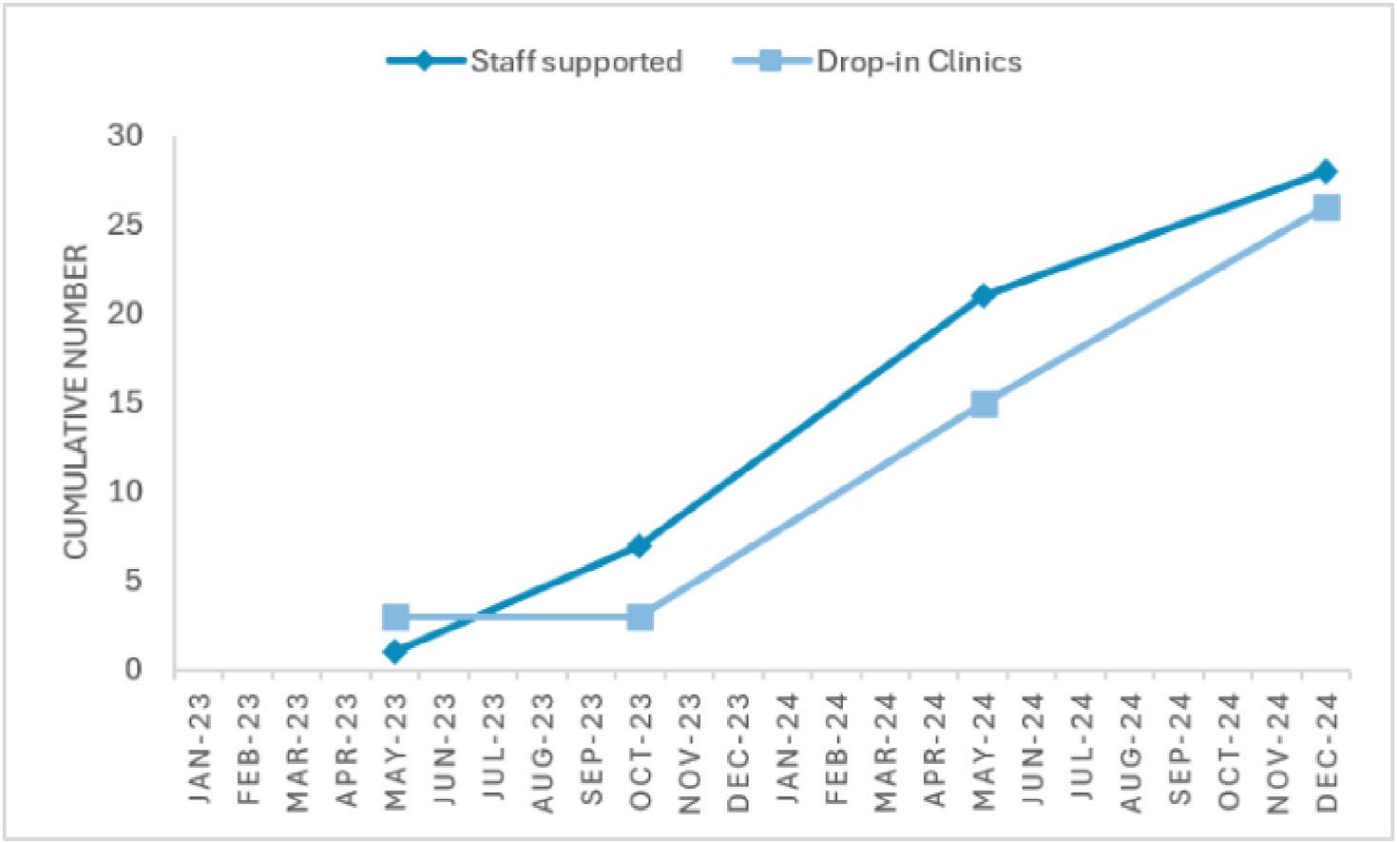
The number of drop‐in clinics offered and number of staff who attended sessions.

### 3.1 Number of People Seeking Information (Objective 1)

With increasing number of sessions (Figure 2), we had increasing numbers of people seeking information, totalling 61 clinicians throughout the data collection period. Attendees were from across NMAHP professional groups (Table 1).

**TABLE 1:** Number of clinicians (per profession) seeking advice from the research support sessions.

Profession	Number of clinicians seeking advice	
	2023	2024
Physiotherapist	2	22
Dietician	2	8
Nurse	2	15
Occupational Therapist	2	6
Midwife	0	1
Radiographer	0	1
**Total**	**8**	**53**

### 3.2 Type and Amount of Support Provided (Objective 2)

We offered increased quantity of support in 2024 in comparison to 2023 (Figure 3). By far the most common type of advice provided was general information about the different funding schemes and signposting to websites for further information. This was followed by application review which was offered to all clinicians intending to submit applications. PPIE and finance advice were accessed less frequently by comparison but were key areas that were subsequently commented on during review of applications review. A total of 37 h were scheduled for planned drop‐in sessions (virtual and face to face). A further 146.2 h were spent supporting people, totalling 183.2 h (Figure 4).

**FIGURE 3:**
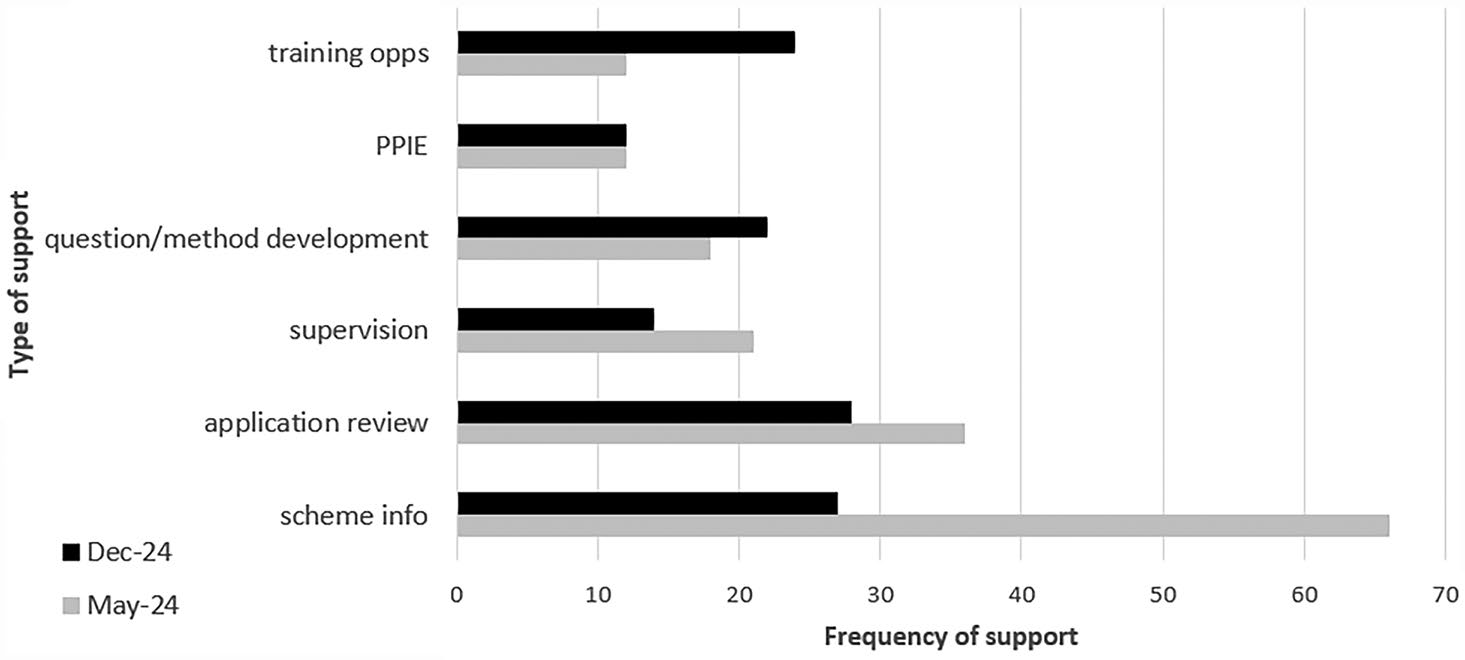
Frequency and focus of support provided by research capacity and capability building team to potential applicants. Abbreviations: PPIE, patient and public involvement and engagement.

**FIGURE 4:**
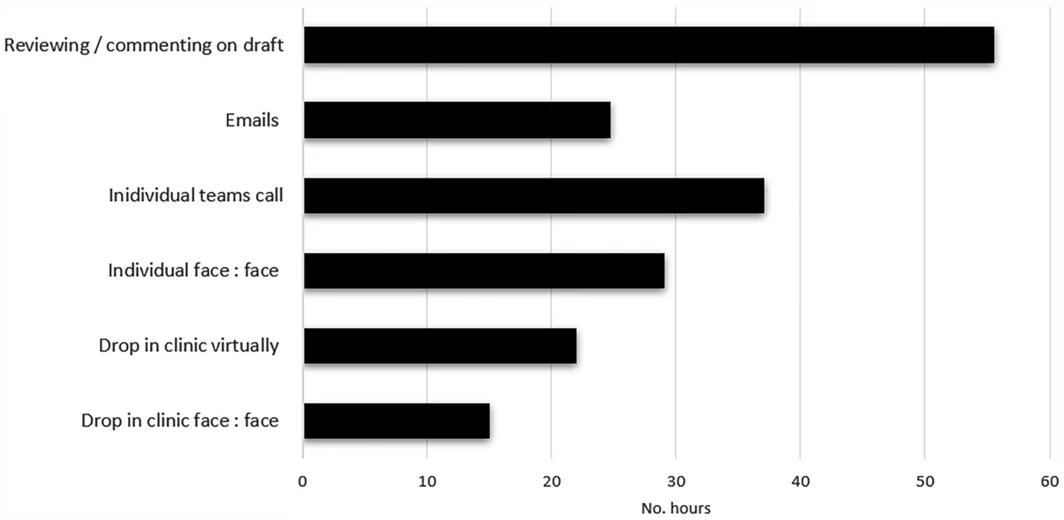
Time (hours) and method of support provided by research capacity and capability building team to potential applicants.

### 3.3 Application Development, Submission and Income Generation (Objective 3)

Over the 18 month period the research support team supported the development of 33 applications across funding calls (Table 2). Of these submissions, 25 (76%) were successful. In total, £234,579 of personal awards were awarded to support NMAHP professional development (Table 2).

**TABLE 2:** Number of application submissions, successful submissions and associated income.

Application scheme	Number of applications submitted	Number of successful submissions *n* (%)	Income per successful submission (£)	Total income generation (£)
Personal Training Award	8	6 (75)	830	4980
BRC fellowship	4	4 (100)	16,700^a^	67,099
NIHR ICA Internship	21	15 (71)^b^		162,500^c^
**TOTAL**	**33**	**25**		**234,579**

Abbreviations: BRC, Biomedical Research Centre; ICA, Integrated Clinical Academic; NIHR, National Institute of Health and Care Research.

^a^ Funding amount varied per applicant. Mean funding per applicant was £16, 700.

^b^ Of the 15 fundable applications, one was deemed fundable but not offered a place and two clinicians were also offered the BRC fellowship and accepted this award instead. There were 12 successful awards commenced.

^c^ One applicant was employed substantively within a HEI, so the funding did not come into the organisation. A total of 11 funded awards were therefore received, totalling £162,500.

### 3.4 Staff Support Evaluation (Objective 4)

In total 53 people had met with team members during support sessions. Seven were not sent the survey because they were not personally applying to schemes (*n* = 3), attended a session for a different reason (*n* = 3) and commenced maternity leave (*n* = 1). The survey was therefore sent to a total of 46 NMAHPs. The overall response rate was 25/46 (54%); 7/20 (35%) physiotherapists, 8/14 (57%) nurses, 6/6 dietitians (100%), 3/5 (60%) (OTs) and 1/1 (100%) midwife.

Prior to the support sessions, staff awareness of funding schemes was low, with over half (*n* = 14, 56%) reporting they were ‘not aware’ of the schemes. Eight (32%) respondents reported having ‘some awareness’ and only 3 (12%) respondents reported being ‘fully aware’ of funding schemes.

All staff were ‘satisfied’ or ‘very satisfied’ with the support they received from the team. For those individuals who had sought advice and not applied for any funding scheme (*n* = 9), multiple reasons were provided (Table 3).

**TABLE 3:** Reported barriers for application development and submission (*n* = 9).

Reason for not progressing application^a^	Frequency
The timing was not right for me	6
I did not have sufficient time to complete the application to the standard I wanted	4
I could not apply due to service needs/managers concerns	2
I could not identify appropriate supervision in time	2
I could not obtain costings in time	2
The scheme was not right for me	4
I could not complete training/education plan in time	1

^a^ Multiple responses allowed per clinician.

Clinicians reported a high interest in applying for future research schemes, with a mean score of 9.08 (Scale 1–10). All respondents ranked the provision of organisationally provided NMAHP support to develop research ideas as ‘highly important’. Regarding further investment for NMAHP support, respondents ranked this as ‘important’, with a mean score of 4.7 (Scale of 1–5).

Eighteen people provided free text feedback about their personal experiences of navigating research opportunities. These aligned to the primary drivers: lack of awareness of schemes, challenge of understanding schemes, and the complexity of application processes and the value of dedicated support to support applications and achieve successful outcomes.

‘Their support has been vital for my application. I had no idea about this scheme prior to meeting ** and they were instrumental in encouraging me to apply in the first place and then supporting me during the application process.’ (Nurse, May 2024).

Drop‐in sessions were seen as valuable to help reach busy clinicians and help explain schemes and the application process.

‘HCPs working in clinical areas need face/face (in person or online) input to “decode” the information about awards available. There is too much reliance on doing a mass email out with links to relevant websites with info on it… What HCPs need is a person to explain the jargon and talk to about the specifics.’ (Dietitian, December 2024).

Dedicated support was cited as a major step to help address deficits in research activity for NMAHP professions.

‘This support is invaluable for AHP's with no knowledge of how to move into clinical research. The specialties underserved in research will remain so without it.’ (Dietitian, December 2024).

## 4 Discussion

This QI project aimed to increase NMAHP awareness and engagement with research development opportunities. By providing dedicated research support sessions delivered by experienced research‐active clinicians, we observed substantial increases in engagement, application submissions and success rates, far exceeding our initial goal of two submissions within an eighteen‐month period.

National strategies for NMAHPs (NHSE 2021, 2023; HEE 2022) emphasise the importance of research engagement and building clinical academic capacity. Our project aimed to establish a community of research‐engaged NMAHPs, recognising that a Clinical Academic Network offers peer support, mentoring opportunities and a forum to promote research schemes (Shepherd and Quinn 2024). Reviewing 2022 application data revealed minimal awareness or engagement with schemes, supporting the decision to focus initially on entry‐level opportunities as a gateway to building a research‐engaged workforce. While initiatives such as Chief Nurse Fellow schemes have demonstrated success (Bramley et al. 2018; Shepherd et al. 2021), these require internal funding for backfill and training. Given substantial NIHR investment in NMAHP research (National Institute Health and Care Research (NIHR) 2023), focusing on early‐career opportunities was timely and strategic.

Clinical academic careers face a workforce crisis, with numbers declining across medicine (Wise 2024) and the aspirational target of 1% of NMAHPs in clinical academic roles remaining unmet since 2016 (AUKUH 2016). In response, the Office for the Strategic Coordination of Health Research (OSCHR) convened Task and Finish groups to explore barriers and opportunities for clinical academic careers, first in medicine (UKRI 2025a) and later specifically for NMAHPs (UKRI 2025b). National ambitions now aim to increase NMAHP clinical academics by 50% over the next 5 years through flexible career pathways, supportive environments, adequate funding and improved workforce monitoring. Our work addresses foundational steps by supporting staff to take their first steps on the clinical academic ladder.

Barriers to clinical academic progression are well documented, particularly securing funding, obtaining organisational support and being released to undertake research opportunities (Avery et al., 2022; Oulton et al. 2022; Carrick‐Sen et al. 2019; Richardson et al. 2019; Dickinson et al. 2017; Newton et al. 2017). Our project addressed many of these barriers but also highlighted lack of awareness as a key challenge. Raising awareness should start from induction (Menzies et al. 2023; Teixeira et al. 2025) and continue through effective signposting, visible organisational support and active managerial encouragement of research development. Visibility of clinical academics is crucial, as aspiring to roles that are not visible is challenging (Bernhardt et al. 2023).

While published accounts of research capacity‐building vary in metrics and scope, reported successes are often modest, such as nine successful applications over 5 years (Bernhardt et al. 2023) or 10 in 5 years (Gerrish and Chapman 2017). In contrast, our project achieved 25 awards within 2 years, reflecting strong staff enthusiasm for dedicated support. Building on this success, future work will target funded Masters awards through the INSIGHT programme for those less than 5 years post‐registration (NIHR 2024d), NIHR pre‐application support fund applications (NIHR 2024e) and pre‐doctoral submissions (NIHR 2024f).

Strengths of this project include prospective data collection on all support offered, allowing comparisons across time points and demonstrating the impact of focused support on engagement and funding success. Evaluating all participants' experiences, not just successful applicants, provided insights into barriers preventing submission and helped set realistic expectations.

Limitations include the single‐centre nature of this QI project, which may limit generalisability, particularly to primary care or social care settings. The support team provided sessions without additional funding, utilising time within existing clinical academic or SRL job plans, which is not available in all organisations. While data were recorded contemporaneously, minor inaccuracies may exist regarding session content.

### 4.1 Act

In summary, this work confirms the value of targeted support for NMAHPs to increase research engagement and funding success. Addressing awareness, accessibility and structured mentorship is critical to developing the next generation of clinical academics and our results have provided valuable information to support our plans to scale up dedicated support. This project demonstrates that focused interventions can provide a strong foundation for long‐term capacity‐building in NMAHP research.

### 4.2 Recommendations for Future Research

Success with funded fellowships and awards for NMAHPs is an important first step to addressing the current crisis in research engaged clinicians (UKRI 2025b) and to meet the NHS Long term plan (NHSE 2025b). However, further research is required to evaluate whether support at this early stage contributes to successful progression of individuals and sustained research engagement. The NIHR ICA Internship programme has been replaced with the NIHR Health and Care Professionals Internship (NIHR 2025). The scheme has a wider remit, including integrating research evidence into practice, research culture, and research delivery staff career development, as well as continuing to support clinical academic development. Further research is required to explore if the revised scheme helps to address national priorities for staff engagement, delivery, and leadership of research (NHSE 2021).

## 5 Conclusion

Through dedicated support we have increased awareness of NMAHP funding schemes, increased application development, submission and successful awards. Consideration is needed regarding the implementation of such a support model to include recurring financial support and resource development. Additionally, scalability to other settings is an important future consideration to ensure the growth in research accessibility of opportunity is equitable across professions and health care settings.

## Author Contributions

**J. C. Menzies:** conceptualization, investigation, writing – original draft, methodology, visualization, writing – review and editing, formal analysis, project administration, data curation, supervision, resources. **H. Pluess‐Hall:** methodology, validation, writing – review and editing, resources, project administration, investigation. **C. Meek:** formal analysis, data curation. **V. Sriram:** conceptualization, writing – review and editing, supervision. **E. Swingwood:** conceptualization, investigation, writing – original draft, methodology, validation, visualization, writing – review and editing, formal analysis, project administration, resources.

## Funding

The authors have nothing to report.

## Ethics Statement

Not required. This project was registered as a service evaluation as per Trust guidance.

## Conflicts of Interest

The authors declare no conflicts of interest.

## Supporting information


**Data S1:** Categories of support.
**Data S2:** Staff evaluation survey questions.

## Data Availability

The data that support the findings of this study are available on request from the corresponding author. The data are not publicly available due to privacy or ethical restrictions.
