# Supplementary material for: Targeted Support for Nursing, Midwifery and Allied Health Professional Research Funding Within an Acute UK NHS Trust: A Brief Report
Source: Nurs Open. 2026 Nov 1;13(11):e70982. doi: 10.1002/nop2.70982 (PMC13724296; doi:10.1002/nop2.70982)
Supplement: Supplementary file 1 [file NOP2-13-e70982-s001.docx]

**Supplementary e-file 1: Categories of support**

a. Scheme information- covering information about the different schemes, eligibility criteria, timelines.
b. Question/ method development- covering the applicant's area of interest and potential methods to address this.
c. Training opportunities- information about different training opportunities that could be accessed and what might be appropriate for their method or personal development.
d. Supervision- information about the role of a supervisor, supporting individuals to identify and approach a potential supervisor.
e. PPIE- information about what PPIE was and how this could be incorporated within their project.
f. Finance- guidance on the funding envelope available, gaining salary costings, funding management for during research awards.
g. Application review- time spent reviewing and commenting on a draft application prior to submission.
h. Developed and submitted applications and associated outcome.

The measures identified were selected because they needed to be simple and quick for the team to record. The team were often running sessions independently of each other, so measurements of time and coding to seven distinct topics of focus, were objective and easy for the team to reliably complete. Inter-rater reliability was reviewed at the start of using the categories (October 2023) by two team members attending the same support session and independently coding the advice provided on two separate occasions. There was 100% agreement on the categories of advice covered.

**Supplementary e file 2: staff evaluation survey questions**

1. I am a… (free text response for profession)
2. I predominantly work at ______ site (drop down boxes for all Trust sites)
3. What was your awareness of the NIHR ICA scheme before you saw the adverts for support sessions? (drop down options: fully aware of the scheme and dates for submission, aware of the scheme but not when it was open to applications, not aware of the scheme at all, not applicable)
4. What forms of contact and support did you receive? Please tick all that apply (options: drop in clinic face to face, drop in virtual clinic, individual face to face meeting, individual virtual meeting, individual emails)
5. How satisfied were you with the help and support provided by the team? (drop down options: very satisfied, satisfied, neither satisfied or dissatisfied, dissatisfied, very dissatisfied)
6. What was the outcome of your attendance/ contact? (options: I applied for a BRC fellowship, I applied for an NIHR ICA Internship, I applied for a personal training award, I did not apply for any of the schemes (reasons explored below), I started to apply but did not complete the application process (reasons explored below), I submitted an application and was successful, I submitted an application but was not invited to interview, I submitted an application but was unsuccessful)
7. If you did not apply for the scheme please tick all the reasons that apply. (Options: not supported by manager, the scheme was not right for me, the timing was not right for me, I did not have sufficient time to complete the application to the standard I wanted, I could not identify appropriate supervision within the time, I could not obtain costings within time ;I could not complete training / education plans within time)
8. How interested are you in applying for future research schemes? (Scale 1-10, with 10 the highest)
9. How important do you think it is that the Trust provides dedicated help and support for Nurses/Midwives/ Allied Health Professionals (NMAHPs) to develop their own research ideas? (Scale 1-5; 5 very important)
10. Do you think more investment is required to help and support NMAHPs to develop their own research ideas? (Scale 1-5, 5 very important)
11. (Optional) If you have any further comments about your experience and why you did or did not apply please share comments below.
